# Case of Fracture of the Spine

**Published:** 1870-10

**Authors:** Daniel T. Nelson

**Affiliations:** Prof. Physiology, etc., Chicago Medical College


ARTICLE V. — *Case of Fracture of the Spine. Patient surviving seventy-two days.* By DAN'L T. NELSON, Prof. Physiology, etc., Chicago Medical College.

June 12, 1869—Was called to see T. M. who had just fallen from near the third floor of a dwelling house in process of erection, through an open stairway to the first floor, striking the second floor in his descent.

The only injuries to indicate the direction of the fall was a slight bruise over the superior border of the occipital bone, and on the left thigh, neither of which were severe, as he evidently struck smooth surfaces—floors or timbers.

The patient complained of pain in all of the extremities, but most in the cervical region of the spine. All power of motion was lost except in the cervical region and above, and here it was prevented by the excessive pain produced.

A careful examination of the spine, in bed, revealed, at the seat of the greatest pain, a separation of the spinous processes of the first and second dorsal vertebræ to about three times their normal distance. No fracture could be detected. This was the only injury noticed, except the bruises referred to above. Below the point of injury there was complete paralysis and loss of sensibility except the pain referred to the extremities.

The patient was an Irish laborer, 23 years of age, muscular, full fleshed, five feet eight inches high, weighing about 150 lbs., and apparently in perfect health at the time of the accident. Prof. N. S. Davis also saw the patient the day of the accident.

On the day of the injury the only medicine given was a mixture containing Morphine and Belladonna to relieve the pain. The attendants were directed to keep the patient all of the time on his sides or chest, and not on the back, to give the injured parts the benefit of gravity.

2nd day. Pulse, 74; respiration, 17. Some delirium, but slept the most of the night.

3rd day. Pulse, 62; respiration, 16. No fever; no delirium.

4th day. Pulse, 65; respiration, 17. Considerable œdema of left arm after lying on that side a part of the night. Slept well.

6th day. Œdema left arm, same. Has not lain on it since 4th day. Pulse, 72; respiration, 18. Pulse fuller, and temperature of skin increased; face flushed—some fever. Tr. Veratrum Viride given until nausea produced; then stopped.

7th to 12th day. Pulse, 60—68; respiration, 16, 17. No Veratrum given, but instead—

| | | |
|---|---|---|
| ℞. | Tr. Digitalis, - - - - - - - - - - - | ʒ j. |
| | Fluid Ext. Ergot, - - - - - - - - - - - | ʒ jv. |
| | Syr. Simplicis, - - - - - - - - - - - | ℥ jss. |
| | Tr. Aurantii Cort., - - - - - - - - - | ʒ ij. |

M. Teaspoonful every six hours.

12th day. Tongue begins to be furred for the first time since the accident. Bowels only move by the assistance of laxatives. Urine, normal in quantity and appearance, acid, and has been so from the first. Drawn every eight hours; appetite good; digestion well performed.

13th to 20th day. Pulse 65—78; respiration, 16—19. Left arm still swollen, but not as much as on 4th day. Tongue only slightly furred; bowels moved almost daily by laxatives or enemas; fæces normal; urine normal; appetite good. Food used daily, since the 7th day, five to six eggs, beef tea from two lbs. beef, bread and butter, tea and fruit; digestion well performed; fever ephemeral, only lasting a few minutes.

Since the 13th day, the following mixture has been given instead of the Digitalis and Ergot of the 7th day:

| | | |
|---|---|---|
| ℞ | Fluid Ext. Belladonnæ, - - - - - - - - - | ʒ j. |
| | Syr. Simplicis, } Aq. Puræ, } - - - - - - - - - - - | āā ℥ ss. |

M. Teaspoonful every four hours.

21st day. Had a chill from no assignable cause, fever followed.

22nd day. Pulse, 90; respiration, 23; not labored. Belladonna stopped, and a mixture containing in each dram Hydrarg. Chlor. Corros. gr. 1-16, and Tr. Cinchona ʒj. Teaspoonful every eight hours.

23rd day. Had a sinking spell.

24th day. The œdema which has never left the arm, began to show itself in the walls of the chest and lower limb of the left side.

26th day. On the suggestion of Dr. Walter Hay who saw the patient in consultation, the Bromide of Mercury was given instead of the Bichloride, with the hope of better quieting the muscular spasms in the limbs and the parts below the seat of injury, which produced pain referred to the seat of injury and above.

℞. Hydrarg. Bromid. - - - - - - - gr. ij.
Sacch. Albi, - - - - - - - - - ʒ j.
M. Ft. chart. No. viij. One powder every eight hours.

30th day. Œdema increasing, both lower extremities swollen. Has invaded left lung and the lower and posterior portion of right, so as to interfere very considerably with respiration. The patient was kept upon the sides and anterior portion of the body during the first ten days, but afterwards, for fear of bedsores and to gratify the patient, he was allowed to lie a part of the time upon the back, but later he was compelled to lie upon the left side with the head considerably raised, on account of the œdema of the left lung and lower lobe of the right.

At the suggestion of Dr. Hay, the Bromide of Mercury was omitted and the Bromide of Iron given instead, as the gums were getting tender and a red line forming next the teeth; the tongue also was red and tender, and the epithelium thinned. Appetite not so good. Urine purulent for the first time since the accident.*

℞. Ferri Bromid., - - - - - - - ʒ j.

Syr. Simplicis, }

Aq. Puræ, } - - - - - - - āā ℥ ij.

M. Teaspoonful every six hours.

* Urine, immediately after being drawn, was found to contain numerous *bacteria*, which, after repeated investigations, were traced to the atmosphere, and to partially desiccated pools of stagnant water in the street gutters. W. H.

Strychnia was also given, with the hope of stimulating the nerves and reducing the œdema.

| | | |
|---|---|---|
| ℞. Strychniæ, | - - - - - - - - - | gr. j. |
| Tr. Aurantii Cort., | - - - - - - - - | ℥ ij. |
| Syr. Symplicis, } Aq. Font., } | - - - - - - - | āā ℥ vij. |

M. Teaspoonful every six hours.

31st day. Take Strychnia every four hours, unless spasms in limbs return.

32nd day. Appetite better; œdema less; urine abundant—two quarts in eighteen hours.

33rd day. Pulse, 90; tongue dry; skin dry; heat increased; appetite poor.

34th day. Pulse, 84; tongue dry, but cleaner.

35th to 38th day. Pulse, 80—76; tongue clean; appetite improving.

39th day. Tongue clean; appetite good; bowels moved of themselves for the first time since injury. Give strychnia every eight hours.

41st day. Capillary circulation of nose and right cheek impeded; spasms of muscles of mastication, producing much pain; quieted by local pressure of hands of attendants. Intellect dull.

42nd day. Strychnia omitted; hyperæsthesia left ear and cheek following the course of the facial nerve—not the *fifth;* spasmodic contraction of muscles of face left side; also of shoulders and abdomen. Pulse, 78; respiration, 19; urine, two quarts; has been abundant since 31st day. Œdema much lessened; respiration easy; line of sensation three inches above right nipple, four inches above on left side of arm to insertion of deltoid anteriorly, not so low on posterior portion, and thence to lower border of scapula. No record of the exact line of sensation was before made, but it had not perceptibly changed since the accident. From the time of the accident until the muscular spasms on the 41st day, there had been hyperæsthesia along the line of sensation above, extending as high as the lower jaw and nearly to the ear, but not reaching it. After the spasms of these muscles the zone of hyperæsthesia passed upward to include the portions of the face below the eyes, the nose, cheeks and ear, leaving the zone below with nearly normal sensibility. The spasms and hyperæsthesia were at first confined to left side, but on the

43rd day the right side also suffered. The hyperæsthesia rendered the muscular spasms almost unendurable. Reflex muscular contractions easily produced in legs on touching them, as in placing a bottle to catch urine. Bedsores clean and healthy, very little discharge. (Have no note of the time when they began, but it was about the 20th day when the sloughs began to show signs of forming. They were kept constantly bathed with a strong solution of Permaganate of Potash, so that there was no odor of decomposing pus in the room, although the weather the most of the time was very warm (July and August.) Muscles of mastication left stiff by spasms; cannot open mouth; sordes begin to collect on teeth; urine, two and a half quarts; pulse, 78, full and strong; respiration, 20.

The dissection made by the bedsores along the spine revealed a fracture of the 7th dorsal vertebra, not before discovered; all the symptoms and the complaints of the patient had directed attention to the injury higher up.

44th day. Pulse, 84; respiration, 19; urine two quarts, and passed for the first time involuntarily but without the catheter; does not dribble but all passed at once after the bladder has become moderately filled; does not feel the urine flowing, but does the *fæces;* refers the sensation to the seat of injury in the neck. Urine began to be purulent on the 30th day, and amount of pus increased until about the 40th day, when it had reached some two ounces in quantity, after which it gradually decreased but rarely was entirely absent until death.

46th day. Pulse, 94; respiration, 27. Feet cold most of the time. Bedsores healthy, and filling up.

48th day. Line of sensation as low as cartilages of lower ribs and ensiform cartilage, right leg almost to toes, both legs to knees, right arm to elbow, left nearly to elbow.

(Can hardly believe now, that sensation did return in lower extremities, but had no doubt of it at the time the record was made.) Feel sure there was no mistake about the extension of sensibility in the chest and arms, as this was observed by Dr. Hay also. Bowels moved of themselves; urine, two quarts. Pulse, 72; respiration, 18.

49th day. Sensation to knees and elbows; urine, two quarts.

50th day. Pulse, 90; respiration, 19; sensation not as low; sleepy; intellect dull; skin hot; delirium for a few minutes; face flushed during delirium; œdema, which has never been entirely absent, is increasing again.

51st day. Dr. Hay, who lives near, was called in last evening. Found pulse, 120; respiration, 36, and difficult; œdema much increased; both lungs dull; mucous rales; respiratory murmur only heard in front above the nipples.

Dr. H. increased the Strychnia, which had been nearly suspended of late—entirely while the spasm of the muscles of mastication had been so troublesome—to gr. 1-16, every four hours, until three doses were taken, and then every six hours.

In the morning, pulse, 102; respiration, 27*; less labored. Continue Strychnia unless spasm of muscles increased.

52nd day. Sensation to second rib and middle of deltoid. Pulse, 102; respiration, 23; slept half of night; urine, two quarts. Œdema decreasing.

53rd day. Pulse, 90; respiration, 24. Hyperæsthesia, lower boundary, the clavicles. Œdema decreasing.

56th day. Pulse, 105; respiration, 27; œdema lower limbs, increasing. Urine alkaline for the first time.

58th day. Hyperæsthesia near clavicles, less. Capillary circulation in cheeks, sluggish.

63d day. Pulse 90; respiration easy. Abscess in left thigh broke and discharged one quart of pus and sanies; femur exposed six inches.

64th day. Pulse, 90; respiration, 34, labored.

68th day. Abscess in left arm burst; said to have discharged more than the one in the thigh; mostly a sanious fluid, like pure juice, or like pus and pure juice mingled. Urine dribbles away; bowels loose.

72nd day. Urine, two quarts. No marked change, though gradually failing; and on the 73d day died.

All the muscles of the left side seemed to be different, much as if putrefaction had already considerably advanced. No autopsy could be obtained on account of the objections of friends.

* Pulse, 96; respiration, 24. W. H.

REMARKS.—We notice, as peculiarities of the case:

1st. The unusual length of time which the patient lived after an injury so high up.

2nd. The slight variation of the pulse and respiration from the normal standard up to the 51st day, when fluids in the lungs had so accumulated as to readily account for their rapidity.

3rd. The long time which the urine remained normal in its reaction, not being alkaline until the 56th day, although pus began to appear as early as the 30th day.

4th. The ease with which digestion and assimilation are performed; showing the possibility of the performance of these functions without the aid of the spinal cord or its nerves below the cervical.

5th. The physiological action of Strychnia in reducing the œdema and increasing the urine, as seen by its exhibition in decided doses on the 30th and following days, and on the 51st and subsequent days.

6th. The good effect of Permang. Potash in preventing profuse suppuration of the bedsores, and keeping away all disagreeable odors, although in the midst of summer.

7th. The location of the line of sensibility as noted on the 42nd day, remembering the anatomical distribution of the nerves in this region, and their origin.

Of the accuracy of the observations there can be no doubt, as it was noticed by Prof. J. A. Allen and Moses Gunn, at different times, and by Dr. Hay and myself at a number of examinations.

8th. The change in the zone of hyperæsthesia, noted on the 41st and 43rd days, and its return to its lower position, as noted on the 53rd day.

May we suppose there was an effusion of fluid into the spinal canal or within the membranes of the cord, which from its pressure destroyed the function of the posterior columns and thus verified the experiments of Brown-Sequard, (see Physiol. and Pathol. Nervous Centres, pp. 19 et seq.) and that the amount of this fluid increased and thus changed the position of this zone as noted?

9th. The extension of sensibility, as noted the 48th day.

May we suppose the real seat of injury to the cord itself was only at the fracture discovered the 43rd day, and the fluids effused from their pressure destroyed the functions of the cord as high as the cervical region, and then, there being a partial absorption of this fluid, the function of the cord partially returns, as noted on the 48th day?

